# Synthesis, crystal structure and properties of poly[(μ-2-methyl­pyridine *N*-oxide-κ^2^ *O*:*O*)bis­(μ-thio­cyanato-κ^2^ *N*:*S*)cobalt(II)]

**DOI:** 10.1107/S2056989023010721

**Published:** 2024-01-01

**Authors:** Christian Näther, Inke Jess

**Affiliations:** a Institut für Anorganische Chemie, Universität Kiel, Max-Eyth.-Strasse 2, 24118 Kiel, Germany; University of Kentucky, USA

**Keywords:** synthesis, crystal structure, layered structure, IR spectrum, thermal properties

## Abstract

The title compound, [Co(NCS)_2_(C_6_H_7_NO)]_*n*_ or Co(NCS)_2_(2-methyl­pyridine *N*-oxide), was prepared by the reaction of Co(NCS)_2_ and 2-methyl­pyridine *N*-oxide in methanol. All crystals obtained by this procedure show reticular pseudo-merohedric twinning, but after recrystallization, one crystal was found that had a minor component with only a very few overlapping reflections. The asymmetric unit consists of one Co^II^ cation, two thio­cyanate anions and one 2-methyl­pyridine *N*-oxide coligand in general positions. The Co^II^ cations are octa­hedrally coordinated by two O-bonding 2-methyl­pyridine *N*-oxide ligands, as well as two S- and two N-bonding thio­cyanate anions, and are connected *via* μ-1,3(*N*,*S*)-bridging thio­cyanate anions into chains that are linked by μ-1,1(*O*,*O*) bridging coligands into layers. No pronounced directional inter­molecular inter­actions are observed between the layers. The 2-methyl­pyridine coligand is disordered over two orientations and was refined using a split model with restraints. Powder X-ray diffraction (PXRD) indicates that a pure sample was obtained and IR spectroscopy confirms that bridging thio­cyanate anions are present. Thermogravimetry and differential thermoanalysis (TG-DTA) shows one poorly resolved mass loss in the TG curve that is accompanied by an exothermic and an endothermic signal in the DTA curve, which indicate the decomposition of the 2-methyl­pyridine *N*-oxide coligands.

## Abstract 2

In the crystal structure of the title compound, the cobalt cations are octa­hedrally coordinated by two N- and two S-bonding thio­cyanate anions as well as two O-bonding 2-methyl­pyridine *N*-oxide coligands. The cations are linked by pairs of anionic ligands into chains that are connected into layers by two μ-1,1-bridging O atoms of the 2-methyl­pyridine *N*-oxide coligands.

## 1. Chemical context

Investigations on the synthesis, structures and properties of coordination compounds is still an active field in coordination chemistry because of their versatile structural behavior and their diverse physical properties. Based on simple considerations concerning the coordination behavior of the cations, the anions and the additional coligands, their structures can be influenced to some extent. In this regard, coordination polymers are of special inter­est, because mono-, di- and tri-periodic networks can be generated, which can show, for example, cooperative magnetic phenomena, non-linear optical properties or conductivity (Yue & Gao, 2019 ▸; Ferrando-Soria *et al.*, 2017 ▸; Wang *et al.*, 2012 ▸; Coronado *et al.*, 2000 ▸). In this context, numerous compounds based on small-sized anions such as, for example, cyanide, azide or carboxyl­ate have been reported in recent years (Nowicka *et al.*, 2012 ▸; Yue & Gao, 2019 ▸; Ohba & Ōkawa, 2000 ▸; Zhou *et al.*, 2012 ▸).

In our own research, we are focused on the synthesis, structures and properties of transition-metal thio­cyanate coordination polymers, for which predominantly chain and layered compounds are observed (Näther *et al.*, 2013 ▸). In most chain compounds, the metal cations are linked by pairs of μ-1,3-bridging thio­cyanate anions and the geometry of the chain – linear or corrugated – depends on the actual metal coordination, *e.g*. all-*trans* or *cis*–*cis*–*trans* (Jochim *et al.*, 2018 ▸; Rams *et al.*, 2020 ▸; Böhme *et al.*, 2020 ▸). For layered compounds, two different motifs are mainly observed in which the metal cations are linked by only single bridging anionic ligands (Werner *et al.*, 2015*a* ▸) or in which two metal cations are connected by pairs of thio­cyanate anions into dinuclear units that are further linked into layers by single μ-1,3-bridging anionic ligands (Werner *et al.*, 2015*b* ▸). Compared to this, thio­cyanate compounds with more condensed networks are rare (Neumann *et al.*, 2018 ▸).

In recent work, we mainly focused on monocoordinating coligands that in nearly all cases consist of pyridine derivatives, but it is noted that chain or layered thio­cyanate networks can be further connected if bridging coligands such as pyrazine or bi­pyridine derivatives are used (Real *et al.*, 1991 ▸; Adams *et al.*, 2010 ▸).

Another class of inter­esting ligands are represented by pyridine *N*-oxide ligands, for which many structures are reported. In nearly all compounds, these ligands show two different coordination modes, which include the terminal N-coordination or the μ-1,1(*O*,*O*)-bridging mode, where the latter mode is of inter­est for the synthesis of compounds with more condensed networks. We therefore became inter­ested in this class of ligands. Within this project, we tried to prepare compounds based on Co(NCS)_2_, which is of inter­est for our project. We used 2-methyl­pyridine as the ligand, for which only three compounds with Mn, Cd and Zn are reported (see *Database survey*). Within this work, we reacted Co(NCS)_2_ with 2-methyl­pyridine in methanol, which led to the formation of violet-colored, block-like crystals, which were characterized by single-crystal X-ray diffraction. This proved that a coordination polymer with the composition Co(NCS)_2_(2-methyl­pyridine *N*-oxide) was obtained that is isotypic to its Cd and Mn analogs already reported in the literature (Mautner *et al.*, 2016 ▸ and 2018 ▸). 
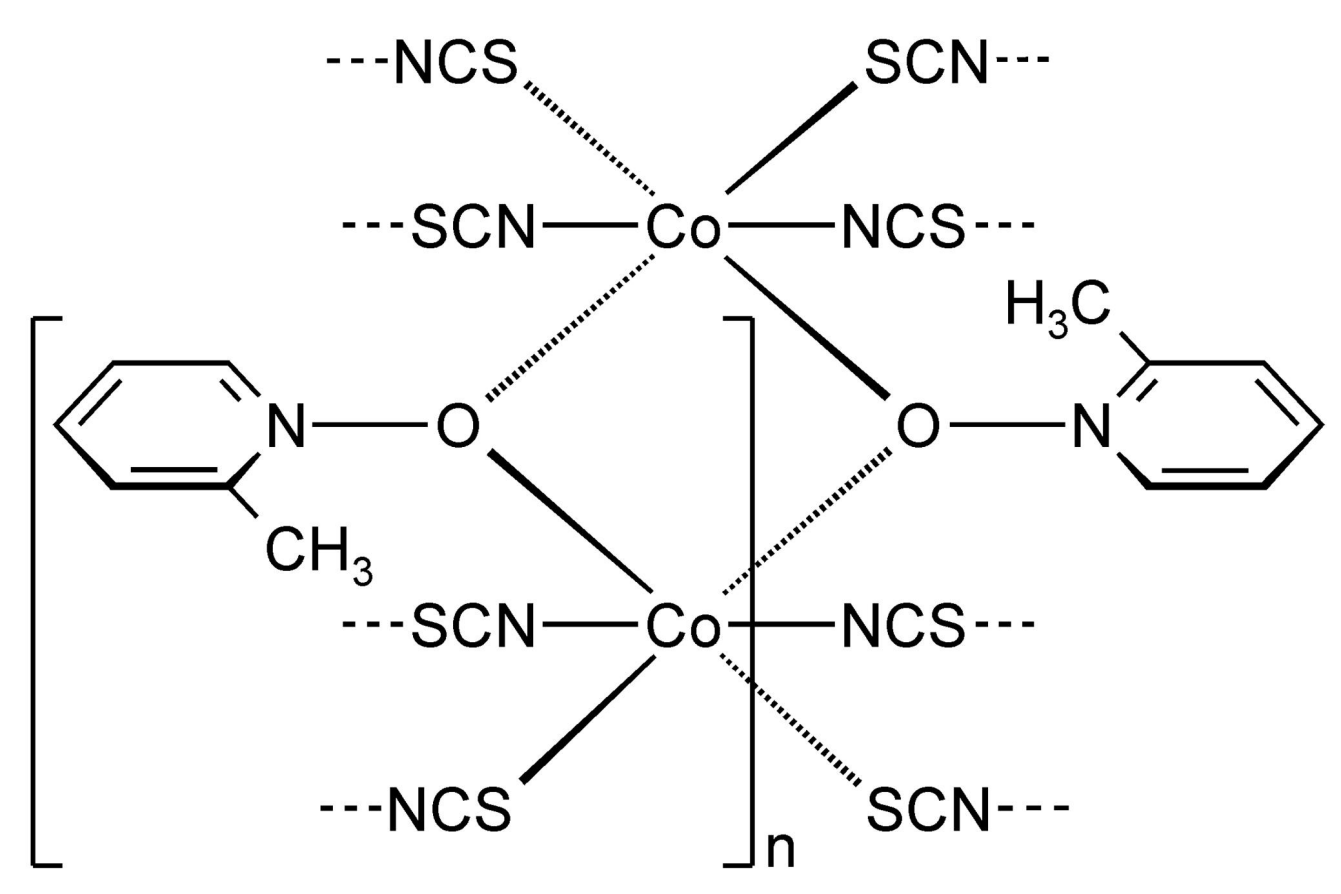


## 2. Structural commentary

**Figure 1:**
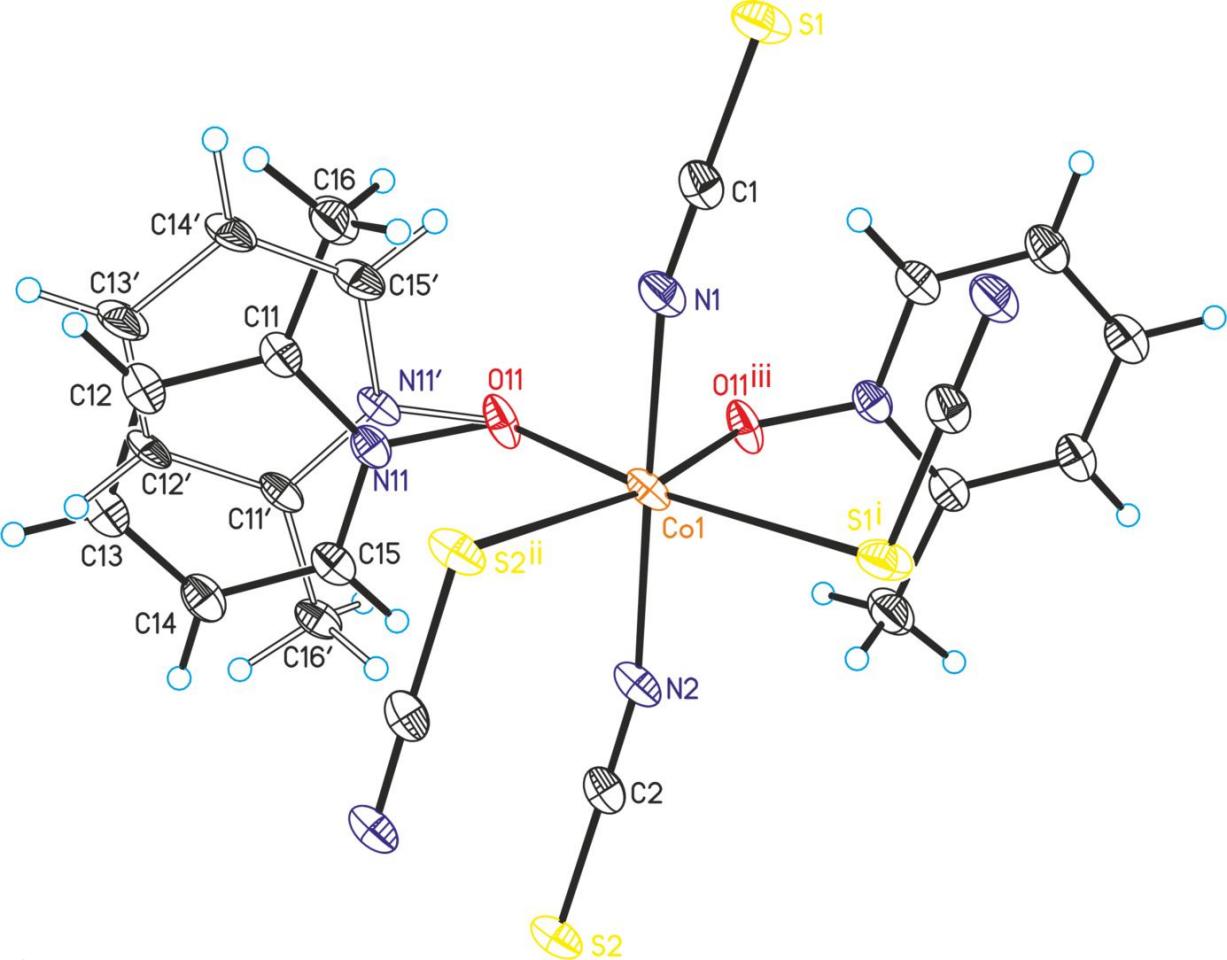
Crystal structure of the title compound showing the Co coordination with labeling and displacement ellipsoids drawn at the 50% probability level. Symmetry codes: (i) −*x* + 1, −*y* + 2, −*z* + 1; (ii) −*x*, −*y* + 1, −*z* + 1; (iii) −*x* + 1, −*y* + 1, −*z* + 1. For clarity, the disorder of the 2-methyl­pyridine *N*-oxide coligand is shown with full and open bonds for only one ligand.

**Figure 2:**
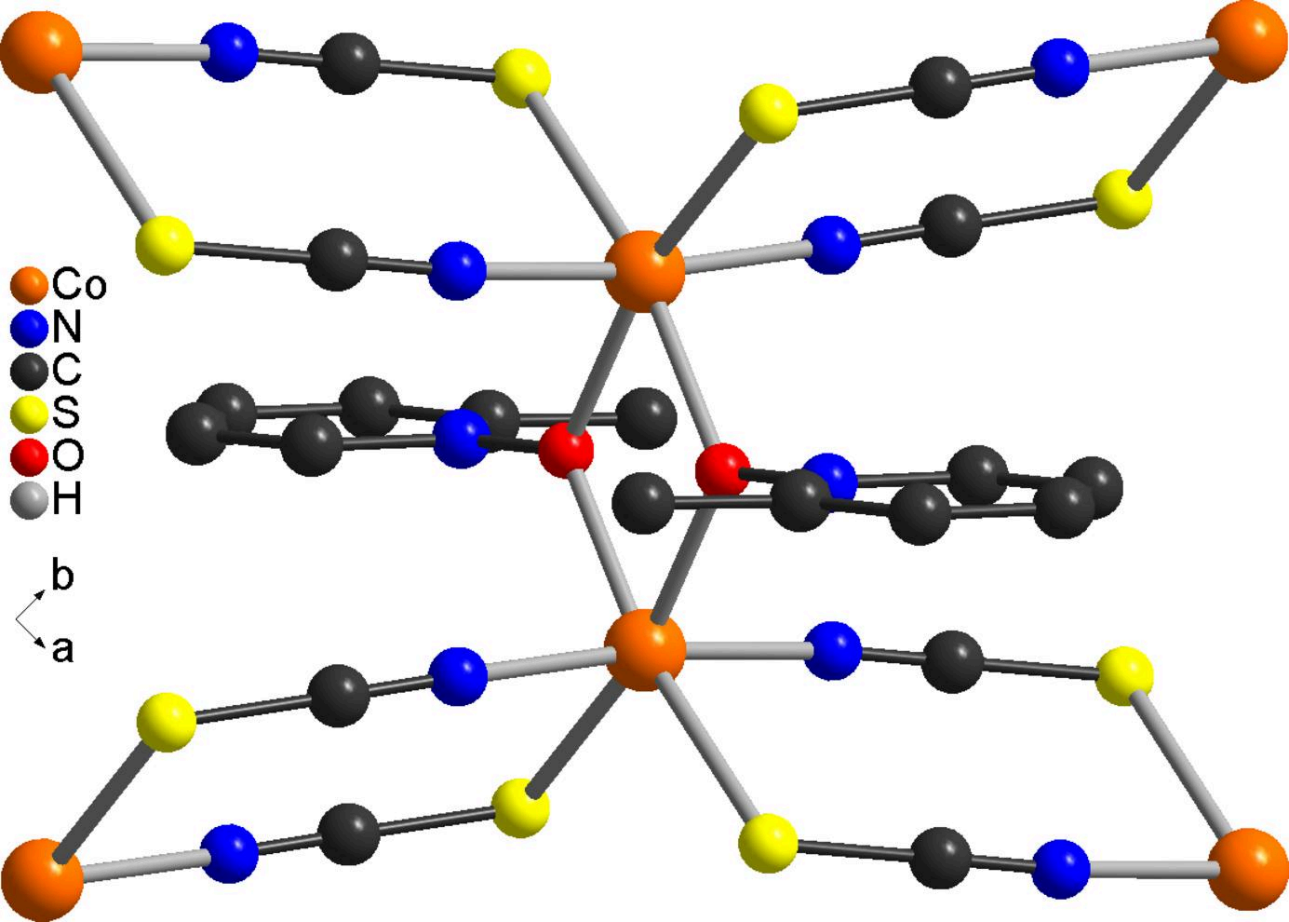
Crystal structure of the title compound, showing the connection of the Co cations. The C—H hydrogen atoms as well as the disorder of the 2-methyl­pyridine *N*-oxide coligand are omitted for clarity.

**Figure 3:**
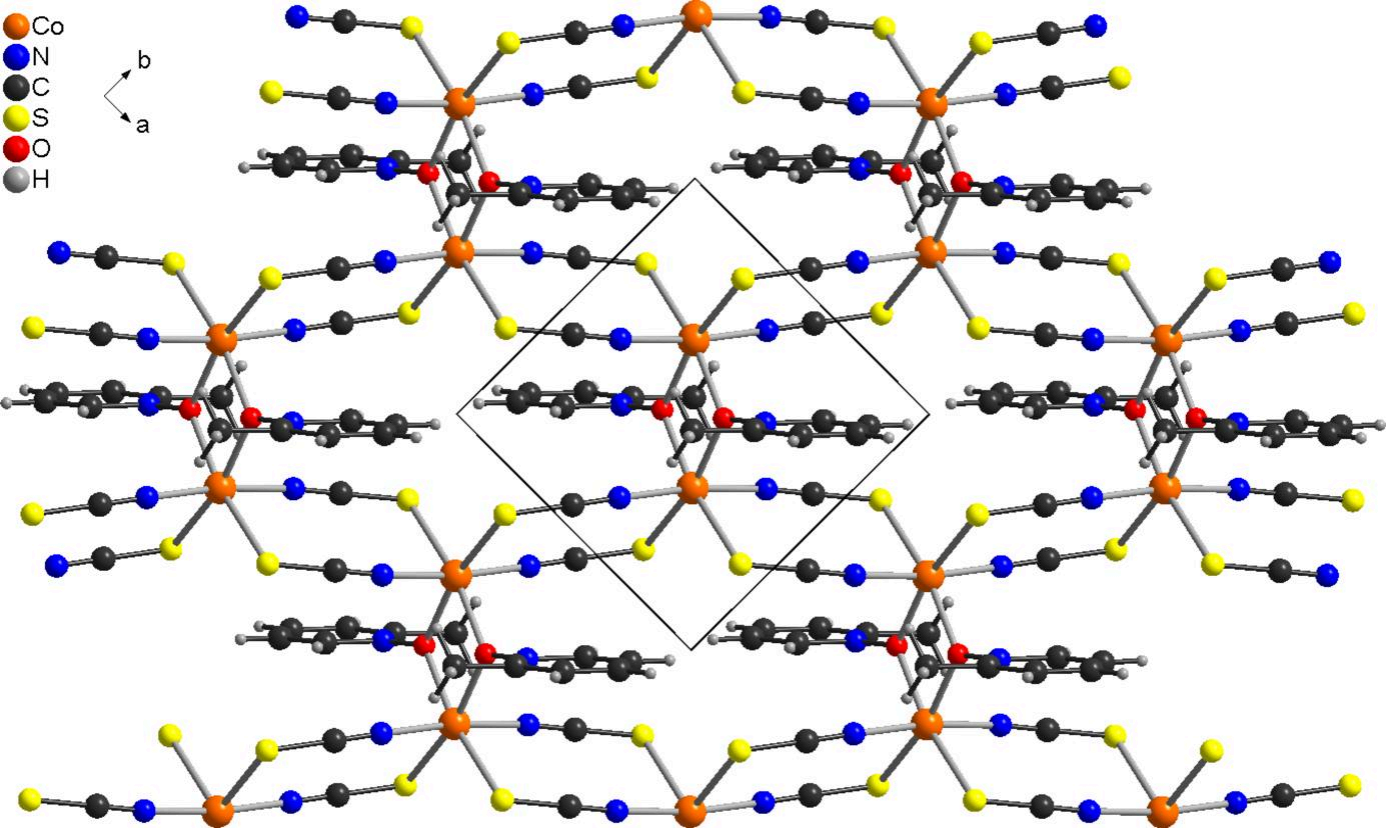
Crystal structure of the title compound with view along the crystallographic *c*-axis direction of a part of a layer. The disorder of the 2-methyl­pyridine coligand is not shown.

The asymmetric unit of the title compound, Co(NCS)_2_(2-methyl­pyridine *N*-oxide), consists of one cobalt cation, one 2-methyl­pyridine *N*-oxide coligand as well as of two thio­cyanate anions, all of them located in general positions (Fig. 1 ▸). The 2-methyl­pyridine coligand is disordered over two orientations and was refined using a split model with restraints (see *Refinement*). The Co^II^ cations are sixfold coordinated by two O atoms of two symmetry-equivalent μ-1,1(*O*,*O*)-bridging 2-methyl­pyridine *N*-oxide coligands, as well as by two N and two S atoms of four μ-1,3(*N*,*S*)-bridging thio­cyanate anions (Fig. 1 ▸). The two N-bonding thio­cyanate anions are in *trans*-positions, whereas the two S-bonding anions as well as the two O atoms of the coligands are in *cis*-positions (Figs. 1 ▸ and 2 ▸). The bond distances correspond to standard values and from the bonding angles it is obvious that the Co^II^ cations are in a slightly distorted octa­hedral coordination (Table 1 ▸). The Co^II^ cations are linked by pairs of thio­cyanate anions that are located on centers of inversion into chains that proceed along the [110] direction (Figs. 2 ▸ and 3 ▸). Because of the *cis*–*cis*–*trans*-coordination, these chains are corrugated with a dihedral angle of 108.52 (3)° between the two neighbouring Co(NCS)_2_Co planes. These chains are further linked into layers by planar four-membered centrosymmetric Co_2_O_2_ rings, built up of two cobalt cations and two μ-1,1(*O*,*O*)-bridging O atoms of the 2-methyl­pyridine *N*-oxide coligands. The layers are parallel to the *ab*-plane (Fig. 3 ▸ and S1).

## 3. Supra­molecular features

In the crystal structure of the title compound, the layers are stacked in the *c*-axis direction and are separated by the 2-methyl­pyridine *N*-oxide coligands (Fig. S1). There are no pronounced directional inter­molecular inter­actions between the layers. There are two inter­layer C—H⋯S and one intra­layer C—H⋯N contacts with large distances and angles far from linearity that should correspond to only very weak inter­actions (Table 1 ▸).

## 4. Database survey

Searching for structures built up of a transition-metal thio­cyanate and 2-methyl­pyridine *N*-oxide using the Cambridge Structural Database (CSD version 5.43, last update November 2021; Groom *et al.*, 2016 ▸) and ConQuest (Bruno *et al.*, 2002) reveals that only three structures have been published. They include *M*(NCS)_2_(2-methyl­pyridine *N*-oxide with *M* = Mn, Cd that are isotypic to the title compound [Refcodes: KESRUY (Mautner *et al.*, 2018 ▸) and UKILIL (Mautner *et al.*, 2016 ▸)]. There is one additional compound with the composition Zn(NCS)_2_(2-methyl­pyridine *N*-oxide)_2_(H_2_O) that is built up of discrete complexes, in which the Zn cations are fivefold coordinated by two terminal N-bonding anionic ligands, two O-bonding 2-methyl­pyridine *N*-oxide coligands and one water mol­ecule (Refcode: UKIMEI; Mautner *et al.*, 2016 ▸).

If the search is extended to compounds with Co(NCS)_2_ and the other methyl­pyridine *N*-oxide isomers, no hits for 3-meth­yl­pyridine *N*-oxide are observed, whereas two structures were found for 4-methyl­pyridine *N*-oxide. The crystal structure of Co(NCS)_2_(4-methyl­pyridine *N*-oxide) is very similar to that of the title compound, because the Co^II^ cations are linked by pairs of thio­cyanate anions into chains that are connected into layers by μ-1,1(*O*,*O*)-bridging O atoms of the 4-methyl­pyridine ligands (Refcode: MEQKOJ; Zhang *et al.*, 2006 ▸). In contrast to the title compound, the N-coordinating thio­cyanate anions are in *cis*-positions, whereas the S-bonding anionic ligands are in a *trans*-configuration. In the second compound, Co(NCS)_2_(4-methyl­pyridine *N*-oxide)(MeOH), the Co^II^ cations are octa­hedrally coordinated by one terminal N-bonding and two bridging thio­cyanate anions, one methanol mol­ecule and two μ-1,1(*O*,*O*)-bridging O atoms of the 4-methyl­pyridine ligands (Shi *et al.*, 2006 ▸). Each of the two Co^II^ cations are linked by pairs of anionic ligands into dinuclear units, which are further connected *via* two μ-1,1(*O*,*O*)-bridging 4-methyl­pyridine ligands into chains.

## 5. Physical characterization

**Figure 4:**
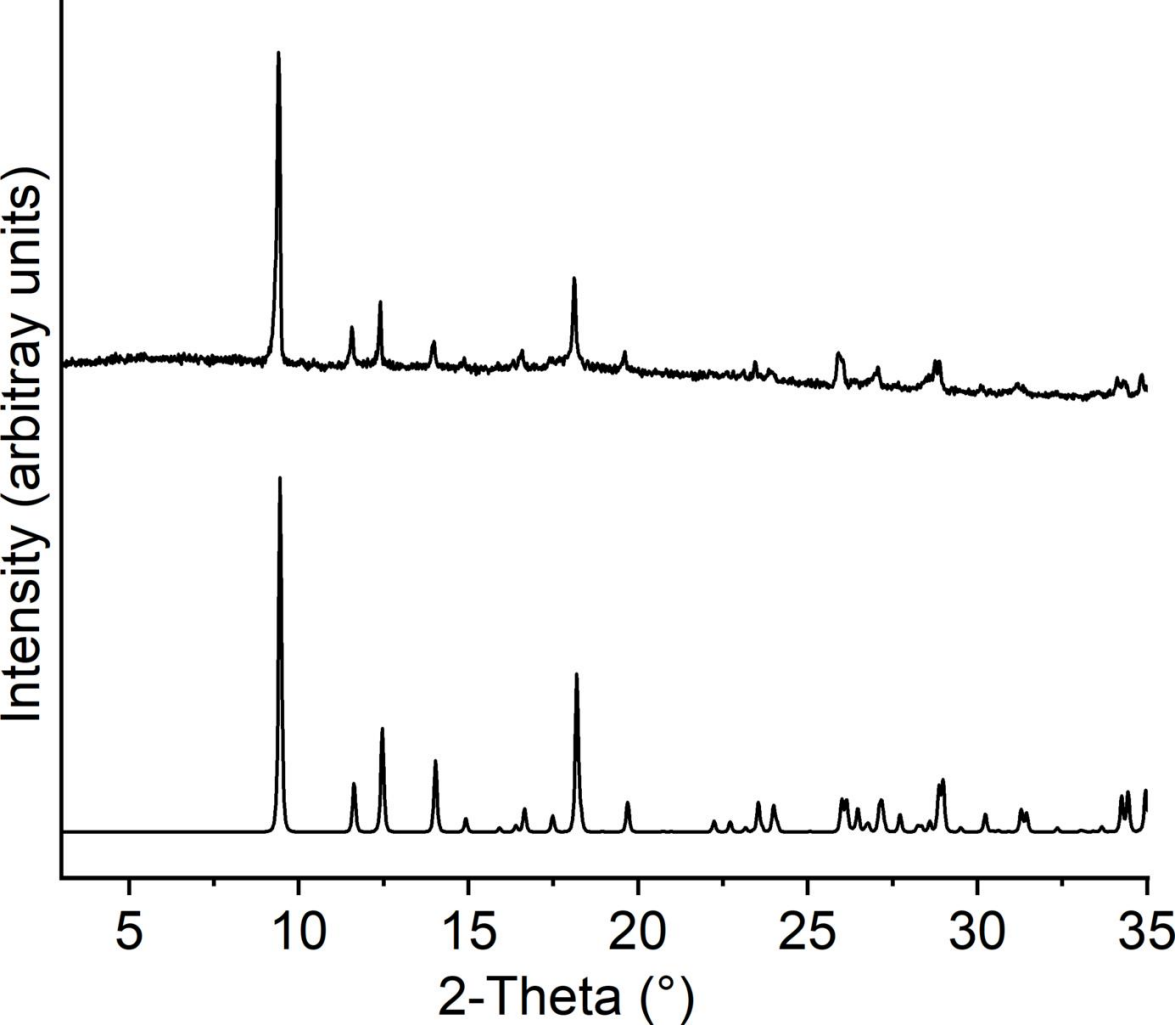
Experimental (top) and calculated X-ray powder pattern (bottom) for the title compound.

Comparison of the experimental X-ray powder pattern with that calculated from single-crystal data proves that a pure crystalline phase has been obtained that is of poor crystallinity (Fig. 4 ▸). In the IR spectrum, the CN stretching vibration is observed at 2107 and 2160 cm^−1^, in agreement with the presence of only μ-1,3-bridging thio­cyanate anions (Fig. S2). The occurrence of two different bands might be traced back to the fact that two crystallographically independent thio­cyanate anions are present.

The thermal properties of the title compound were investigated by thermogravimetry (TG) and differential thermoanalysis (DTA). Upon heating, decomposition starts at about 240°C, followed by a continuous mass loss that is not finished until 450°C (Fig. S3). From the first derivative of the TG curve (DTG curve), it is indicated that this mass loss consists of two different steps, which cannot be resolved even at lower heating rates. The mass loss is accompanied by a small exothermic signal, followed by an endothermic signal, which points to the decomposition of the coligands, as observed in previous work (Näther & Jess, 2023 ▸).

## 6. Synthesis and crystallization

**Synthesis**

Co(NCS)_2_ (99%) was purchased from Sigma Aldrich and 2-methyl­pyridine *N*-oxide (96%) from Aldrich. Single crystals were prepared by reacting 0.5 mmol of Co(NCS)_2_ (87.1 mg), with 0.25 mmol of 2-methyl­pyridine *N*-oxide (27.3) mg in 1 mL of methanol. Within 3 days, violet-colored crystals were obtained that all showed reticular pseudo-merohedric twinning. A nearly single crystal was found in a recrystallized batch. A microcrystalline powder was obtained using the same amount of reactants under continuous stirring.

**Experimental details**

The data collection was performed using an XtaLAB Synergy, Dualflex, HyPix diffractometer from Rigaku with Cu *K*α radiation. The PXRD measurements were performed with a Stoe Transmission Powder Diffraction System (STADI P) with a MYTHEN 1K detector, a Johansson-type Ge(111) monochromator and Cu *K*α_1_ radiation (λ = 1.540598 Å). The IR spectrum was measured using an ATI Mattson Genesis Series FTIR Spectrometer. Thermogravimetry and differential thermoanalysis (TG-DTA) was performed in a dynamic nitro­gen atmosphere in Al_2_O_3_ crucibles using an STA-PT 1000 thermobalance from Linseis. The instrument was calibrated using standard reference materials.

## 7. Refinement

Crystal data, data collection and structure refinement details are summarized in Table 2 ▸. In the beginning, it was noted that all crystals investigated consisted of reticular pseudo-merohedric twins, giving the impression of a very large unit cell axis. For these crystals, about 26% of the reflections overlap (Fig. S4). Several of these twins were measured, which always revealed the same kind of twinning. For these crystals only poor reliability factors were obtained, even if a twin refinement using data in HKLF-5 format were used. After recrystallization from methanol, however, one crystal was found that consisted of two inter­grown individuals and in this case less than 2% of the reflections overlapped if a measurement at large detector distances was performed (Fig. S5). A two-component refinement with data in HKLF-5 format led to comparable reliability factors and, therefore, the data presented here originate from a refinement neglecting the smaller component.

The C-bound H atoms were positioned with idealized geometry (methyl H atoms allowed to rotate but not to tip) and were refined isotropically with *U*_iso_(H) = 1.2 *U*_eq_(C) (1.5 for methyl H atoms) using a riding model. The 2-methyl­pyridine coligand is disordered over two orientations and was refined using a split model with SADI and RIGU as restraints. Refinement of the occupancy led to a value of 0.841 (4) for N11 to C16.

## Supplementary Material

Crystal structure: contains datablock(s) I. DOI: 10.1107/S2056989023010721/pk2699sup1.cif

Structure factors: contains datablock(s) I. DOI: 10.1107/S2056989023010721/pk2699Isup2.hkl

Figure S1. Crystal structure of the title compound with view of the arrangement of the layers along the crystallographic a-axis direction. DOI: 10.1107/S2056989023010721/pk2699sup3.png

Figure S2. IR spectrum of the title compound. Given are the values of the CN stretching vibration of the thiocyanate anions. DOI: 10.1107/S2056989023010721/pk2699sup4.png

Figure S3. TG, DTA and DTG curve of the title compound. DOI: 10.1107/S2056989023010721/pk2699sup5.png

Figure S4. Reciprocal space plot of a crystal of the title compound showing the partial merohedral twinning, which is common for most crystals. Reflections of the second indiviual are indicated in turquoise. DOI: 10.1107/S2056989023010721/pk2699sup6.png

Figure S5. Reciprocal space plot of the crystal of the title compound used for the structure determination. Reflections of the second indiviual are indicated in turquoise. DOI: 10.1107/S2056989023010721/pk2699sup7.png

CCDC reference: 2314409

Additional supporting information:  crystallographic information; 3D view; checkCIF report

## Figures and Tables

**Table 1:** Hydrogen-bond geometry (Å, °)

*D*—H⋯*A*	*D*—H	H⋯*A*	*D*⋯*A*	*D*—H⋯*A*
C14—H14⋯S1^i^	0.95	2.81	3.491 (3)	129
C14—H14⋯S2^ii^	0.95	2.82	3.514 (3)	131
C16—H16*A*⋯N1	0.98	2.63	3.484 (4)	145

Symmetry codes: (i) 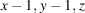 ; (ii) 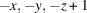 .

**Table 2:** Experimental details

Crystal data	
Chemical formula	[Co(NCS)_2_(C_6_H_7_NO)]
*M*_r_	284.22
Crystal system, space group	Monoclinic, *P*2_1_/*c*
Temperature (K)	100
*a*, *b*, *c* (Å)	7.67386 (11), 7.66462 (9), 18.8755 (2)
β (°)	97.4258 (12)
*V* (Å^3^)	1100.89 (2)
*Z*	4
Radiation type	Cu *K*α
μ (mm^−1^)	15.58
Crystal size (mm)	0.16 × 0.12 × 0.10

Data collection	
Diffractometer	XtaLAB Synergy, Dualflex, HyPix
Absorption correction	Multi-scan (*CrysAlis PRO*; Rigaku OD, 2023 ▸)
*T*_min_, *T*_max_	0.392, 1.000
No. of measured, independent and observed [*I* > 2σ(*I*)] reflections	16436, 2355, 2337
*R*_int_	0.023
(sin θ/λ)_max_ (Å^−1^)	0.637

Refinement	
*R*[*F* ^2^ > 2σ(*F* ^2^)], *wR*(*F* ^2^), *S*	0.029, 0.066, 1.17
No. of reflections	2355
No. of parameters	202
No. of restraints	108
H-atom treatment	H-atom parameters constrained
Δρ_max_, Δρ_min_ (e Å^−3^)	0.37, −0.48

Computer programs: *CrysAlis PRO* (Rigaku OD, 2023 ▸), *SHELXT2014/5* (Sheldrick, 2015*b* ▸), *SHELXL2016/6* (Sheldrick, 2015*a* ▸), *DIAMOND* (Brandenburg & Putz, 1999 ▸) and *publCIF* (Westrip, 2010 ▸).

## supplementary crystallographic information

### Crystal data

**Table:**

[Co(NCS)_2_(C_6_H_7_NO)]	*F*(000) = 572
*M**_r_* = 284.22	*D*_x_ = 1.715 Mg m^−^^3^
Monoclinic, *P*2_1_/*c*	Cu *K*α radiation, λ = 1.54184 Å
*a* = 7.67386 (11) Å	Cell parameters from 12043 reflections
*b* = 7.66462 (9) Å	θ = 4.7–78.3°
*c* = 18.8755 (2) Å	µ = 15.58 mm^−^^1^
β = 97.4258 (12)°	*T* = 100 K
*V* = 1100.89 (2) Å^3^	Block, violet
*Z* = 4	0.16 × 0.12 × 0.10 mm

### Data collection

**Table:**

XtaLAB Synergy, Dualflex, HyPix diffractometer	2355 independent reflections
Radiation source: micro-focus sealed X-ray tube, PhotonJet (Cu) X-ray Source	2337 reflections with *I* > 2σ(*I*)
Mirror monochromator	*R*_int_ = 0.023
Detector resolution: 10.0000 pixels mm^-1^	θ_max_ = 79.1°, θ_min_ = 4.7°
ω scans	*h* = −9→7
Absorption correction: multi-scan (CrysAlisPro; Rigaku OD, 2023)	*k* = −9→9
*T*_min_ = 0.392, *T*_max_ = 1.000	*l* = −23→23
16436 measured reflections	

### Refinement

**Table:**

Refinement on *F*^2^	Hydrogen site location: inferred from neighbouring sites
Least-squares matrix: full	H-atom parameters constrained
*R*[*F*^2^ > 2σ(*F*^2^)] = 0.029	*w* = 1/[σ^2^(*F*_o_^2^) + (0.0149*P*)^2^ + 1.9175*P*] where *P* = (*F*_o_^2^ + 2*F*_c_^2^)/3
*wR*(*F*^2^) = 0.066	(Δ/σ)_max_ = 0.001
*S* = 1.17	Δρ_max_ = 0.37 e Å^−^^3^
2355 reflections	Δρ_min_ = −0.48 e Å^−^^3^
202 parameters	Extinction correction: *SHELXL2016/6* (Sheldrick, 2015a), Fc^*^=kFc[1+0.001xFc^2^λ^3^/sin(2θ)]^-1/4^
108 restraints	Extinction coefficient: 0.00044 (8)

### Special details

**Table:**

Geometry. All esds (except the esd in the dihedral angle between two l.s. planes) are estimated using the full covariance matrix. The cell esds are taken into account individually in the estimation of esds in distances, angles and torsion angles; correlations between esds in cell parameters are only used when they are defined by crystal symmetry. An approximate (isotropic) treatment of cell esds is used for estimating esds involving l.s. planes.

### Fractional atomic coordinates and isotropic or equivalent isotropic displacement parameters (Å^2^)

**Table:**

	*x*	*y*	*z*	*U*_iso_*/*U*_eq_	Occ. (<1)
Co1	0.34308 (4)	0.65702 (5)	0.50034 (2)	0.01618 (11)	
N1	0.4776 (3)	0.8322 (3)	0.44534 (11)	0.0208 (4)	
C1	0.5616 (3)	0.9467 (3)	0.42847 (12)	0.0178 (4)	
S1	0.68342 (7)	1.10993 (7)	0.40518 (3)	0.02297 (14)	
N2	0.1858 (2)	0.5034 (3)	0.55502 (11)	0.0197 (4)	
C2	0.0761 (3)	0.4137 (3)	0.57123 (12)	0.0173 (4)	
S2	−0.07968 (7)	0.28422 (7)	0.59360 (3)	0.02083 (14)	
O11	0.4258 (2)	0.4442 (2)	0.44114 (9)	0.0216 (3)	
N11	0.3245 (4)	0.3636 (4)	0.38733 (13)	0.0169 (5)	0.841 (4)
C11	0.3369 (3)	0.4046 (4)	0.31808 (14)	0.0191 (5)	0.841 (4)
C12	0.2263 (4)	0.3189 (4)	0.26500 (18)	0.0216 (6)	0.841 (4)
H12	0.230783	0.346504	0.216205	0.026*	0.841 (4)
C13	0.1095 (4)	0.1932 (4)	0.28298 (16)	0.0233 (6)	0.841 (4)
H13	0.034696	0.134137	0.246672	0.028*	0.841 (4)
C14	0.1026 (4)	0.1545 (4)	0.35428 (15)	0.0210 (5)	0.841 (4)
H14	0.023386	0.068607	0.367329	0.025*	0.841 (4)
C15	0.2116 (4)	0.2416 (4)	0.40574 (16)	0.0182 (5)	0.841 (4)
H15	0.207596	0.216017	0.454751	0.022*	0.841 (4)
C16	0.4679 (4)	0.5389 (4)	0.30386 (18)	0.0292 (7)	0.841 (4)
H16A	0.440560	0.649386	0.326122	0.044*	0.841 (4)
H16B	0.463728	0.555500	0.252188	0.044*	0.841 (4)
H16C	0.585805	0.500344	0.323906	0.044*	0.841 (4)
N11'	0.3614 (17)	0.3997 (17)	0.3791 (6)	0.012 (2)	0.159 (4)
C11'	0.2342 (16)	0.2832 (16)	0.3592 (6)	0.016 (2)	0.159 (4)
C12'	0.1856 (19)	0.2643 (19)	0.2851 (7)	0.018 (3)	0.159 (4)
H12'	0.095521	0.184123	0.267840	0.021*	0.159 (4)
C13'	0.2677 (19)	0.361 (2)	0.2368 (8)	0.024 (2)	0.159 (4)
H13'	0.233256	0.348303	0.186914	0.029*	0.159 (4)
C14'	0.4001 (18)	0.4778 (18)	0.2616 (7)	0.022 (2)	0.159 (4)
H14'	0.457737	0.544852	0.229290	0.026*	0.159 (4)
C15'	0.4451 (18)	0.4935 (19)	0.3330 (7)	0.021 (2)	0.159 (4)
H15'	0.536599	0.571321	0.350960	0.025*	0.159 (4)
C16'	0.157 (2)	0.1870 (19)	0.4155 (8)	0.018 (3)	0.159 (4)
H16D	0.066740	0.106925	0.393423	0.027*	0.159 (4)
H16E	0.249770	0.120445	0.444436	0.027*	0.159 (4)
H16F	0.104815	0.269982	0.446106	0.027*	0.159 (4)

### Atomic displacement parameters (Å^2^)

**Table:**

	*U*^11^	*U*^22^	*U*^33^	*U*^12^	*U*^13^	*U*^23^
Co1	0.01039 (17)	0.01187 (18)	0.0253 (2)	−0.00181 (13)	−0.00148 (13)	0.00080 (14)
N1	0.0176 (9)	0.0175 (10)	0.0271 (10)	−0.0046 (8)	0.0021 (8)	−0.0025 (8)
C1	0.0133 (10)	0.0160 (11)	0.0233 (11)	0.0019 (9)	−0.0001 (8)	−0.0030 (9)
S1	0.0194 (3)	0.0162 (3)	0.0350 (3)	−0.0040 (2)	0.0102 (2)	−0.0005 (2)
N2	0.0141 (9)	0.0164 (9)	0.0272 (10)	−0.0039 (7)	−0.0029 (7)	0.0035 (8)
C2	0.0148 (10)	0.0139 (10)	0.0214 (11)	0.0020 (8)	−0.0041 (8)	0.0020 (8)
S2	0.0137 (2)	0.0181 (3)	0.0295 (3)	−0.0029 (2)	−0.0020 (2)	0.0083 (2)
O11	0.0142 (7)	0.0193 (8)	0.0286 (9)	0.0002 (6)	−0.0078 (6)	−0.0075 (7)
N11	0.0143 (11)	0.0141 (11)	0.0215 (8)	0.0003 (8)	−0.0010 (6)	−0.0005 (6)
C11	0.0174 (9)	0.0171 (10)	0.0218 (8)	−0.0020 (8)	−0.0008 (6)	−0.0001 (6)
C12	0.0192 (10)	0.0194 (11)	0.0247 (9)	−0.0016 (9)	−0.0025 (8)	−0.0021 (8)
C13	0.0198 (11)	0.0204 (12)	0.0285 (9)	−0.0022 (9)	−0.0022 (7)	−0.0010 (7)
C14	0.0169 (10)	0.0162 (11)	0.0287 (9)	0.0004 (8)	−0.0018 (6)	−0.0011 (7)
C15	0.0141 (9)	0.0138 (10)	0.0260 (9)	0.0009 (8)	0.0001 (7)	0.0003 (7)
C16	0.0287 (12)	0.0295 (13)	0.0283 (15)	−0.0132 (11)	0.0000 (11)	0.0021 (11)
N11'	0.007 (4)	0.004 (4)	0.024 (3)	0.003 (3)	−0.001 (2)	0.003 (2)
C11'	0.011 (4)	0.009 (4)	0.027 (3)	−0.001 (3)	−0.001 (2)	0.001 (2)
C12'	0.013 (5)	0.013 (5)	0.026 (3)	−0.008 (4)	0.000 (2)	0.002 (2)
C13'	0.020 (4)	0.022 (5)	0.031 (3)	−0.012 (3)	0.002 (3)	0.006 (3)
C14'	0.018 (4)	0.018 (5)	0.029 (3)	−0.009 (3)	0.001 (3)	0.008 (3)
C15'	0.016 (4)	0.017 (5)	0.029 (3)	−0.004 (4)	0.001 (2)	0.007 (3)
C16'	0.014 (5)	0.011 (5)	0.029 (3)	−0.001 (4)	0.001 (3)	0.003 (3)

### Geometric parameters (Å, º)

**Table:**

Co1—N1	2.055 (2)	C14—H14	0.9500
Co1—S1^i^	2.5507 (7)	C14—C15	1.371 (4)
Co1—N2	2.057 (2)	C15—H15	0.9500
Co1—S2^ii^	2.5507 (6)	C16—H16A	0.9800
Co1—O11^iii^	2.1126 (16)	C16—H16B	0.9800
Co1—O11	2.1200 (17)	C16—H16C	0.9800
N1—C1	1.158 (3)	N11'—C11'	1.340 (13)
C1—S1	1.654 (2)	N11'—C15'	1.354 (14)
N2—C2	1.158 (3)	C11'—C12'	1.408 (14)
C2—S2	1.650 (2)	C11'—C16'	1.478 (14)
O11—N11	1.346 (3)	C12'—H12'	0.9500
O11—N11'	1.258 (11)	C12'—C13'	1.389 (14)
N11—C11	1.359 (4)	C13'—H13'	0.9500
N11—C15	1.351 (4)	C13'—C14'	1.387 (14)
C11—C12	1.391 (4)	C14'—H14'	0.9500
C11—C16	1.488 (4)	C14'—C15'	1.351 (15)
C12—H12	0.9500	C15'—H15'	0.9500
C12—C13	1.387 (5)	C16'—H16D	0.9800
C13—H13	0.9500	C16'—H16E	0.9800
C13—C14	1.386 (4)	C16'—H16F	0.9800
			
N1—Co1—S1^i^	89.21 (6)	C13—C14—H14	120.4
N1—Co1—N2	173.32 (8)	C15—C14—C13	119.2 (3)
N1—Co1—S2^ii^	86.46 (6)	C15—C14—H14	120.4
N1—Co1—O11^iii^	93.52 (7)	N11—C15—C14	120.5 (3)
N1—Co1—O11	91.85 (7)	N11—C15—H15	119.7
S1^i^—Co1—S2^ii^	103.58 (2)	C14—C15—H15	119.7
N2—Co1—S1^i^	86.86 (6)	C11—C16—H16A	109.5
N2—Co1—S2^ii^	89.22 (5)	C11—C16—H16B	109.5
N2—Co1—O11	93.38 (7)	C11—C16—H16C	109.5
N2—Co1—O11^iii^	91.99 (7)	H16A—C16—H16B	109.5
O11^iii^—Co1—S1^i^	91.66 (5)	H16A—C16—H16C	109.5
O11—Co1—S1^i^	164.49 (5)	H16B—C16—H16C	109.5
O11—Co1—S2^ii^	91.93 (4)	O11—N11'—C11'	128.8 (10)
O11^iii^—Co1—S2^ii^	164.76 (5)	O11—N11'—C15'	107.0 (9)
O11^iii^—Co1—O11	72.83 (7)	C11'—N11'—C15'	124.2 (11)
C1—N1—Co1	165.51 (19)	N11'—C11'—C12'	115.9 (11)
N1—C1—S1	179.3 (2)	N11'—C11'—C16'	118.3 (11)
C1—S1—Co1^i^	104.18 (8)	C12'—C11'—C16'	125.8 (11)
C2—N2—Co1	165.02 (18)	C11'—C12'—H12'	119.6
N2—C2—S2	179.3 (2)	C13'—C12'—C11'	120.8 (13)
C2—S2—Co1^ii^	104.54 (8)	C13'—C12'—H12'	119.6
Co1^iii^—O11—Co1	107.17 (7)	C12'—C13'—H13'	120.1
N11—O11—Co1	124.58 (16)	C14'—C13'—C12'	119.8 (13)
N11—O11—Co1^iii^	126.14 (16)	C14'—C13'—H13'	120.1
N11'—O11—Co1	126.2 (7)	C13'—C14'—H14'	120.8
O11—N11—C11	120.9 (2)	C15'—C14'—C13'	118.4 (12)
O11—N11—C15	116.8 (2)	C15'—C14'—H14'	120.8
C15—N11—C11	122.3 (3)	N11'—C15'—H15'	119.6
N11—C11—C12	118.1 (3)	C14'—C15'—N11'	120.8 (12)
N11—C11—C16	117.8 (2)	C14'—C15'—H15'	119.6
C12—C11—C16	124.1 (3)	C11'—C16'—H16D	109.5
C11—C12—H12	119.8	C11'—C16'—H16E	109.5
C13—C12—C11	120.3 (3)	C11'—C16'—H16F	109.5
C13—C12—H12	119.8	H16D—C16'—H16E	109.5
C12—C13—H13	120.2	H16D—C16'—H16F	109.5
C14—C13—C12	119.5 (3)	H16E—C16'—H16F	109.5
C14—C13—H13	120.2		
			
Co1—O11—N11—C11	98.2 (3)	C11—N11—C15—C14	−0.6 (5)
Co1^iii^—O11—N11—C11	−100.5 (3)	C11—C12—C13—C14	0.5 (4)
Co1—O11—N11—C15	−81.8 (3)	C12—C13—C14—C15	0.1 (4)
Co1^iii^—O11—N11—C15	79.5 (3)	C13—C14—C15—N11	−0.1 (4)
Co1^iii^—O11—N11'—C11'	95.9 (16)	C15—N11—C11—C12	1.2 (5)
Co1—O11—N11'—C11'	−93.1 (16)	C15—N11—C11—C16	−179.0 (3)
Co1^iii^—O11—N11'—C15'	−84.3 (12)	C16—C11—C12—C13	179.0 (3)
Co1—O11—N11'—C15'	86.8 (12)	N11'—C11'—C12'—C13'	0 (2)
O11—N11—C11—C12	−178.8 (3)	C11'—N11'—C15'—C14'	2 (3)
O11—N11—C11—C16	1.0 (4)	C11'—C12'—C13'—C14'	0 (2)
O11—N11—C15—C14	179.4 (2)	C12'—C13'—C14'—C15'	0 (2)
O11—N11'—C11'—C12'	178.2 (13)	C13'—C14'—C15'—N11'	−1 (2)
O11—N11'—C11'—C16'	−2 (2)	C15'—N11'—C11'—C12'	−2 (2)
O11—N11'—C15'—C14'	−177.9 (13)	C15'—N11'—C11'—C16'	178.5 (16)
N11—C11—C12—C13	−1.1 (4)	C16'—C11'—C12'—C13'	−179.7 (15)

Symmetry codes: (i) −*x*+1, −*y*+2, −*z*+1; (ii) −*x*, −*y*+1, −*z*+1; (iii) −*x*+1, −*y*+1, −*z*+1.

### Hydrogen-bond geometry (Å, º)

**Table:**

*D*—H···*A*	*D*—H	H···*A*	*D*···*A*	*D*—H···*A*
C14—H14···S1^iv^	0.95	2.81	3.491 (3)	129
C14—H14···S2^v^	0.95	2.82	3.514 (3)	131
C16—H16*A*···N1	0.98	2.63	3.484 (4)	145

Symmetry codes: (iv) *x*−1, *y*−1, *z*; (v) −*x*, −*y*, −*z*+1.
