# Supplementary figures and images for: Synthesis, crystal structure and properties of poly[(μ-2-methyl­pyridine N-oxide-κ2 O:O)bis­(μ-thio­cyanato-κ2 N:S)cobalt(II)]
Source: Acta Crystallogr E Crystallogr Commun. 2024 Jan 1;80(Pt 1):67–71. doi: 10.1107/S2056989023010721 (PMC10833374; doi:10.1107/S2056989023010721)

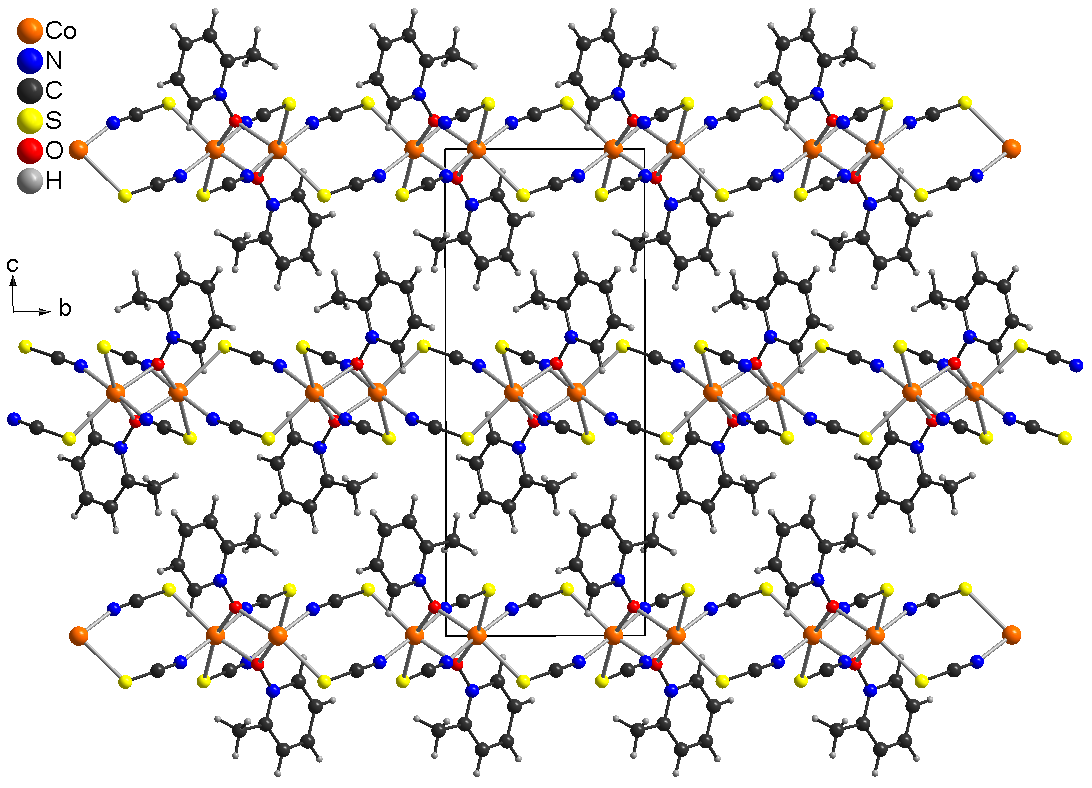

Supplement: Supplementary file 3 [file e-80-00067-sup3.png]

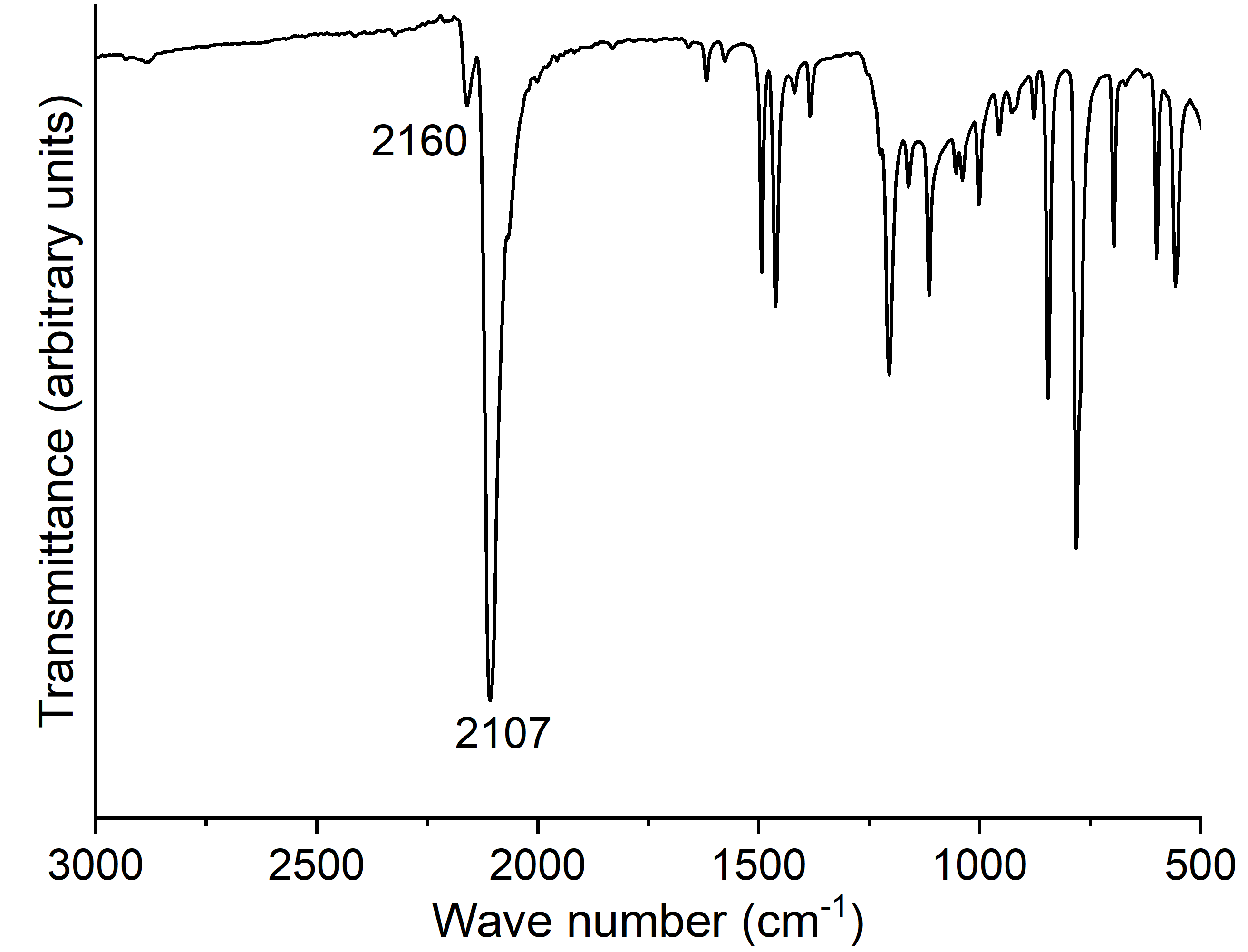

Supplement: Supplementary file 4 [file e-80-00067-sup4.png]

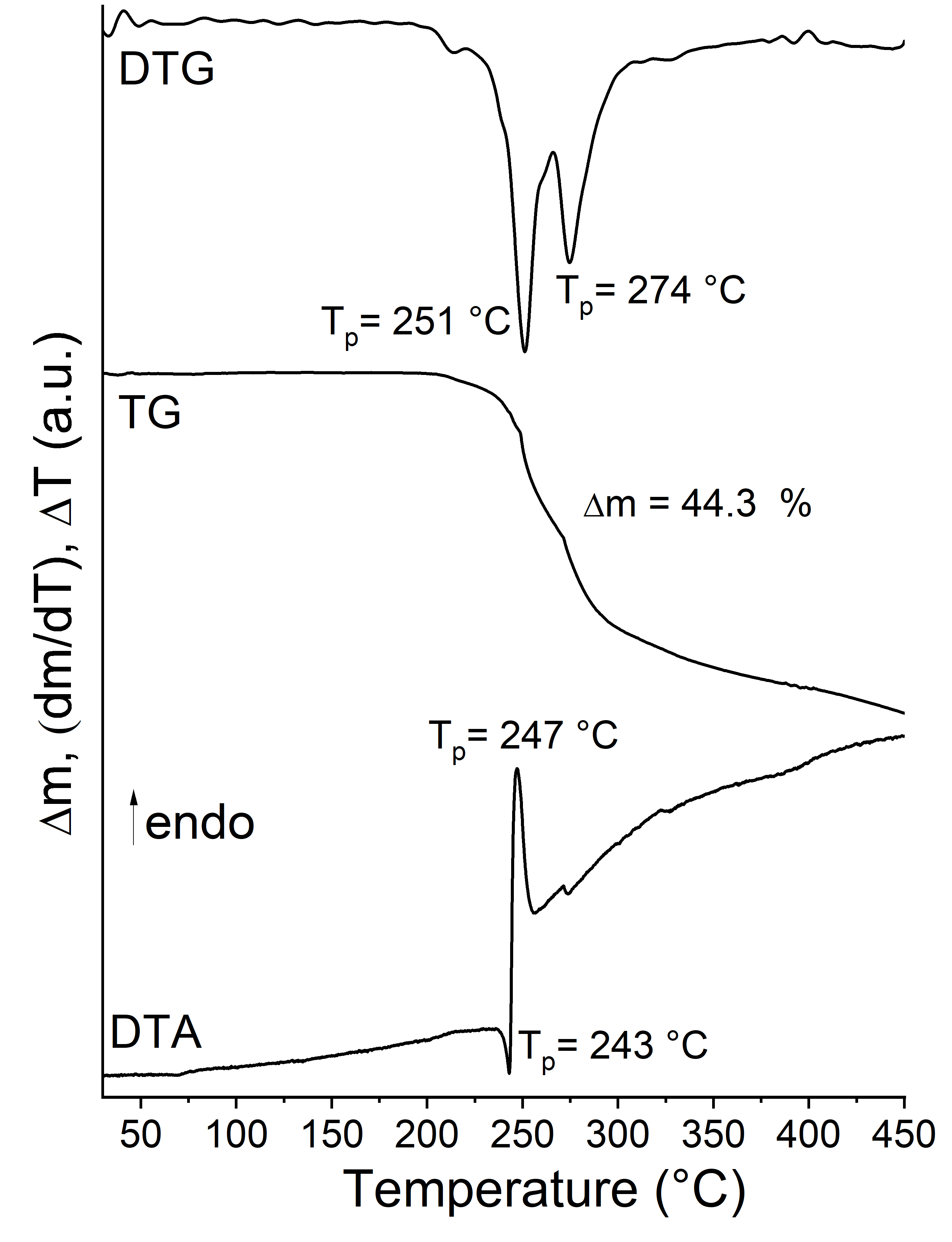

Supplement: Supplementary file 5 [file e-80-00067-sup5.png]

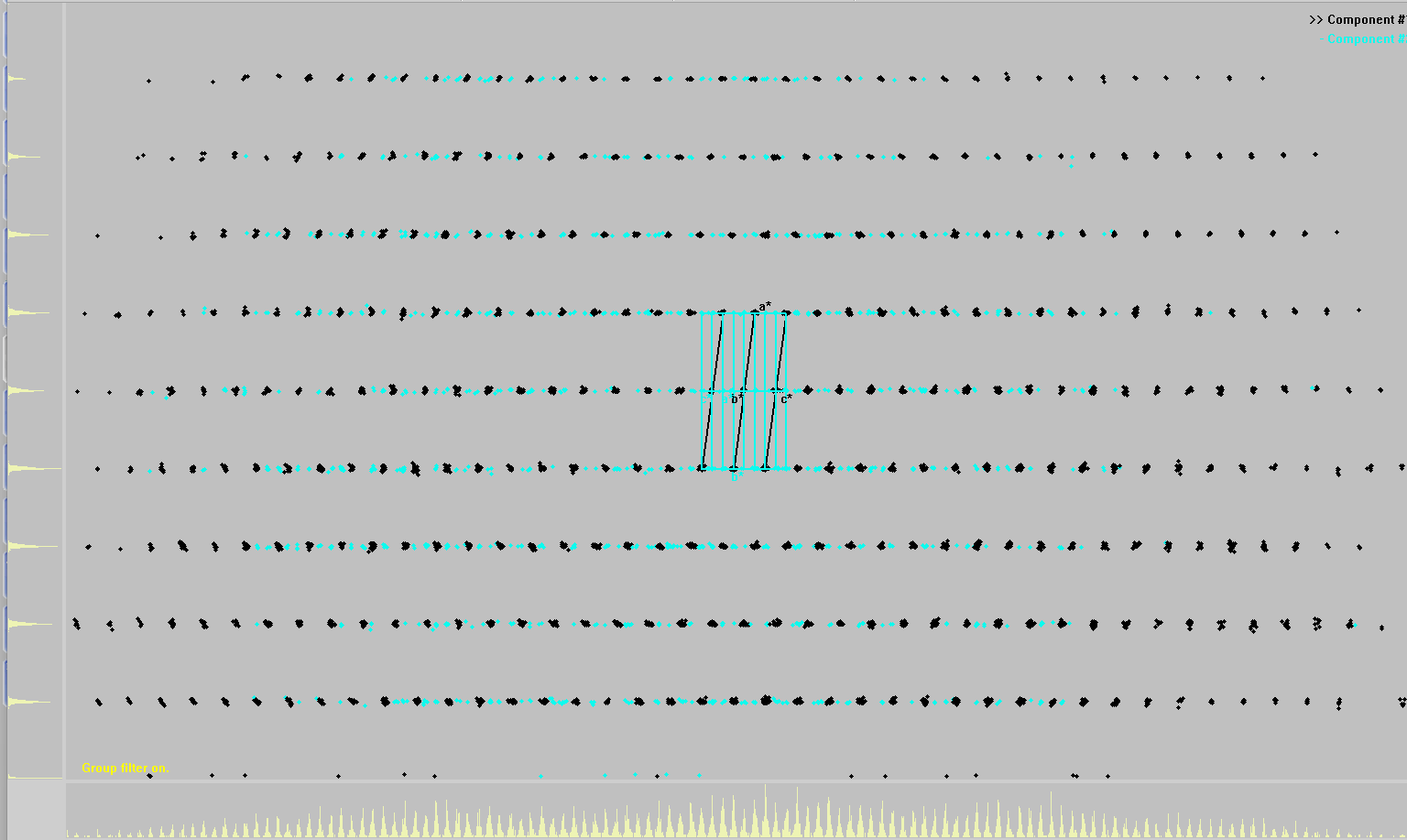

Supplement: Supplementary file 6 [file e-80-00067-sup6.png]

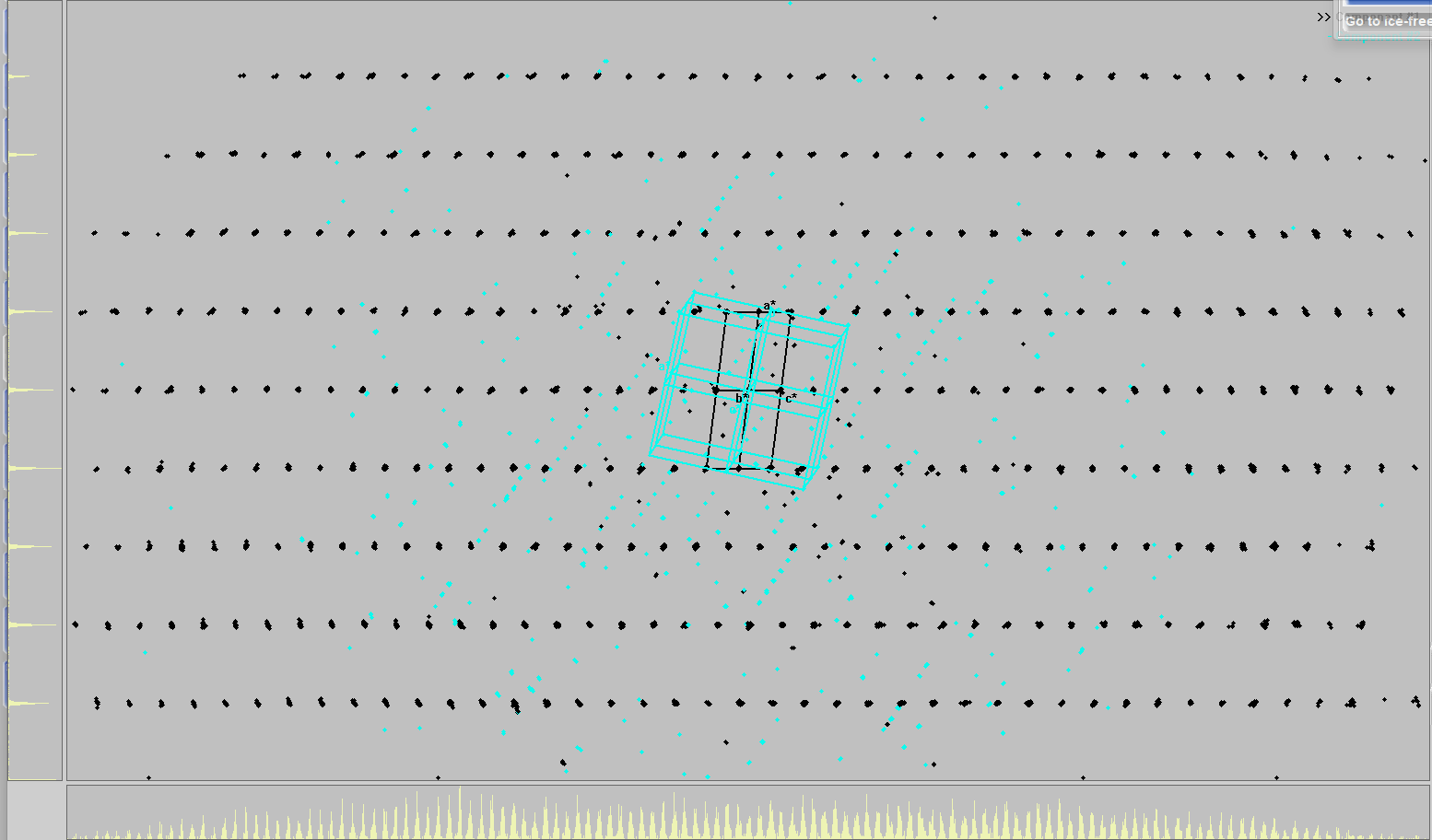

Supplement: Supplementary file 7 [file e-80-00067-sup7.png]
